# Prof. Mendel’s Views of the Therapeutic Value of Hypnotism

**Published:** 1889-10

**Authors:** 


### SELECTIONS.

### Prof. Mendel's Views of the Therapeutic Value of Hypnotism.

Prof. Mendel gave at the same meeting an exposition of his views on the therapeutic value of hypnotism, which was listened to with great attention. Mendel has himself employed hypnotism as a therapeutic agent since 1881, and believes the time has come to formulate definite views on its therapeutic worth. While he does not reject this mysterious agent altogether, he nevertheless insists on a great restriction of its indications. Hypnotism creates an acute morbid disturbance in the functional activity of the cerebral cortex, which, as his own experience has shown, may give rise to very serious disorders. He therefore recommends caution in the employment of hypnotism. He recalls cases in his own practice in which the induction of hypnotism led to serious paroxysms of hysteria and epilepsy, which previously had not existed at all. The frequent exhibition of hypnotism may even lead to what might be properly termed "hypnotismania," *i. e.*, a nervous disturbance which can be relieved only by the constant induction of hypnotism. It can not, however, be denied that in many instances hypnotism has given excellent results, and that by its aid disorders have disappeared which previously yielded to no other treatment. But in all these cases relapses have been so frequent that the benefits derived from this treatment appear after all but of a doubtful nature. We can say, therefore, that hypnotism, though often able to remove the symptoms of a disorder, proves nevertheless seldom a cure. Mendel's belief in the therapeutic power of hypnotism has diminished from year to year, and he can only coincide with the view entertained at present by Charcot, that *hypnotism is worthy of a trial only after all other methods of treatment have failed.*

In this connection I shall relate an interesting little experiment from the domain of hypnotism of which I read some time ago in a medical journal. A German physician was traveling in the cars with two friends of his, one of whom he had formerly cured of a rheumatic affection of the right leg by the aid of hypnotic suggestion. This friend of the physician suddenly falling asleep, the latter endeavored to change his natural sleep into an hypnotic state. He passed his hands very gently three times over the leg of the sleeper, just as he had done previously in the course of his hypnotic treatment, and brought his arm into a horizontal position. The arm remained in that position. The physician then suggested to his friend that the latter was asleep and had to continue sleeping until a certain station was reached. He also suggested to the sleeper that the latter had borrowed of him five dollars which must be returned at the dinner-table with an appropriate apology. The sleeper promised to do every thing as he was bidden. He awoke punctually at the station mentioned, and returned the borrowed money at dinner with a graceful apology. In this case the recollection of the gentle stroking during the treatment had been sufficient to change the physiological sleep into the hypnotic state.

